# Overlap of Miller-Fisher Syndrome and Pharyngeal-Cervical-Brachial Variant Secondary to COVID-19 in Recurrent Guillain-Barré Syndrome: A Case Report

**DOI:** 10.7759/cureus.74954

**Published:** 2024-12-02

**Authors:** Tarek Hammad, Sayeed Hossain, Amin Alayyan

**Affiliations:** 1 Cardiology, Northampton General Hospital, Northampton, GBR; 2 Internal Medicine, East Suffolk & North Essex Foundation Trust, Suffolk, GBR; 3 Internal Medicine, Leicester Royal Infirmary, Leicester, GBR

**Keywords:** covid 19, gillian barre syndrome, miller-fisher syndrome, miller fisher variant, pharyngeal-cervical-brachial (pcb) variant

## Abstract

Miller-Fisher syndrome (MFS) is characterized by the three major components of ophthalmoplegia, ataxia, and areflexia. The occurrence of MFS is relatively uncommon because of its monophasic nature, while recurrent Guillain-Barré syndrome (GBS) is a well-known condition. The pharyngeal-cervical-brachial (PCB) variant is a scarce variant of GBS (3%), which presents with muscle weakness initially involving the neck, oropharynx, and upper extremities. The patient's first symptoms were tingling in all the limbs, followed by ophthalmoplegia, ataxia, and areflexia. Additional bilateral ptosis and flu-like illness were also present. The patient subsequently developed a choking sensation with pharyngeal muscle weakness, which necessitated ventilatory support. Cerebrospinal fluid (CSF) protein levels and anti-ganglioside antibodies were both negative. During the patient's hospital admission, he received intravenous immunoglobulins (IVIGs), indicating that immunomodulating medications may be useful in managing MFS. This constellation of symptoms was induced by SARS-CoV-2 infection, confirmed by a positive polymerase chain reaction (PCR) test. This case underscores the critical role of comprehensive history-taking and physical examination in diagnosing such cases, as COVID-19-induced GBS variants have frequently demonstrated repeatedly negative antibody results. We present an unusual case of a 63-year-old male with MFS induced by COVID-19, with overlapping symptoms of the PCB variant of GBS on a background of previously recurrent GBS.

## Introduction

Guillain-Barré syndrome (GBS) is an acutely progressive autoimmune polyradiculoneuropathy characterized by a progressive ascending pattern of sensory-motor symptoms bilaterally [1]. It often has an infectious inciting event that leads to an autoimmune response. Infectious causes include campylobacter jejuni, cytomegalovirus, Epstein-Barr virus, and human immunodeficiency virus (HIV). Most recently, SARS-CoV-2 infection has been reported widely as a triggering cause [1,2]. It has also been associated with some atypical biochemical and pathophysiological features which can make diagnosis of GBS problematic [3].

In addition to typical sensory-motor symptoms, GBS can present with different signs and symptoms giving rise to variants. These can also include pure sensory or pure motor. Other presentations include ophthalmoplegia, ataxia, and areflexia classically found in the Miller-Fisher syndrome (MFS) variant [1,3]. The signs of MFS combined with decreased consciousness are called Bickerstaff brainstem encephalitis (BBE) [1]. Very rarely, it can also present with weakness in the facial, oropharyngeal muscles, and upper limbs with sparing of the lower limbs, which is classified as the pharyngeal-cervical-brachial (PCB) variant [1,3]. 

The inflammatory stage of this disease is hypothesized to be caused by antibodies (Abs) produced by the immune system intended to neutralize viral or bacterial components. These Abs unfortunately bind to peripheral nerve antigens instead due to a high level of molecular resemblance between certain antigens present on peripheral nerves and the foreign antigens expressed by the causative infectious organism. This process is known as molecular mimicry [4]. This results in demyelination of the nerves and significantly delayed conduction leading to motor and sensory symptoms [4]. This can simultaneously affect breathing and cause respiratory failure. Weakness of the respiratory muscles demands regular spirometry monitoring and early intensive care intervention. When the forced vital capacity drops below 20 mL/kg, mechanical ventilation is advised [5].

MFS is an uncommon variant of GBS which usually presents with a triad of ataxia, areflexia, and ophthalmoplegia [6]. It can also have atypical features such as facial nerve palsy, bulbar palsy hyposthenia, and limb dysesthesia [6,7]. The third, fourth, and sixth cranial nerves are mostly implicated in MFS [6]. Ophthalmoparesis is hypothesized to be caused by anti-GQ1b Abs directly attacking the neuromuscular junction between the cranial nerves and eye muscles [6,8]. Although Abs to the GQ1b ganglioside is a common serological result, the lack of Abs does not entirely exclude the condition [3, 9-10]. MFS is treated like GBS; with supportive care, and intravenous immunoglobulin (IVIG) [6]. MFS has also been reported to overlap with other variants [11,12].

The PCB variant's challenging presentation mentioned earlier can also be confused with other diseases, such as myasthenia gravis or botulism [13,14]. In many studies, PCB has been shown to overlap with MFS [14]. In this case, we present a patient with a previous history of recurrent GBS presenting with features of MFS overlapping with PCB variant secondary to COVID-19 infection.

## Case presentation

A male patient in his sixties presented to Accident and Emergency (A&E) with generalized fatigue and malaise. Two days after his fatigue started, he developed double vision, weakness in the distal muscles of his lower limbs, and tingling and burning sensations in his upper and lower limbs. On the day of his presentation, he tested positive for COVID-19 by reverse transcription-polymerase chain reaction (RT-PCR). On admission, his oxygen saturation was 89% requiring 2 L of oxygen via nasal cannula. The remainder of his vitals were normal. Blood test results on the day of admission, shown in Table 1, revealed an elevated white cell count, neutrophilia, and lymphopenia.

**Table 1:** Blood test results on admission. g, grams; mg, milligrams; mL, milliliters; L, liter; µmol, micromole; mmol, millimole; IU, International Units (IU); sAG, serum antigen; HIV, human immunodeficiency virus; Ab, antibody; Ag, antigen

Blood test (unit)	Result	Reference range
Hemoglobin (g/L)	119	130-170
White cell count (×10⁹/L)	11.2	4.0-10.0
Neutrophil count (×10⁹/L)	9.83	1.5-6.5
Lymphocyte count (×10⁹/L)	0.76	1.1-3.5
Platelet count (×10⁹/L)	316	150-400
International normalized ratio (INR ratio)	1.1	0.8-1.2
C-reactive protein (mg/L)	132	0-5
Estimated glomerular filtration rate (mL/minute)	59	90-120
Creatinine (µmol/L)	95	59-104
Sodium (mmol/L)	137	133-146
Potassium (mmol/L)	4.5	3.5-5.3
Albumin (g/L)	23	35-50
Corrected calcium (mmol/L)	2.48	2.20-2.60
Total bilirubin (µmol/L)	9	0-21
Alanine transaminase (IU/L)	10	5-41
Alkaline phosphatase (IU/L)	67	30-130
Hepatitis B sAg	Negative	N/A
Hepatitis C screen	Negative	N/A
HIV 1 / 2 Ab p24 Ag	Negative	N/A

On examination, the patient was alert and verbalizing but dysphonic. He was hesitant to walk due to sudden loss of balance, and it was decided not to assess his gait for his safety. Further neurological examination revealed bilateral leg weakness with an ascending pattern. The sensation was decreased in both lower limbs. Ankle and knee reflexes were absent bilaterally. Cranial nerve examination revealed bilateral ptosis with diplopia in all directions of gaze. The initial impression following the assessment was GBS with a Miller-Fisher variant, and the plan was to start IVIG for five days and regularly monitor lung function through spirometry to monitor the forced vital capacity.

Over the same day, he developed dysphagia and absent upper limb reflexes, and he started to become more dysphonic and dyspneic. His hourly forced vital capacity decreased from 4.3 to 3.8 L over two hours. Arterial blood gases showed type 2 respiratory failure. Given his worsening respiratory function, he was intubated and sent to the intensive care unit (ICU). It was also noted that his intubation was difficult due to vocal cord paralysis observed by the intensive therapy unit team. These additional rapidly developing bulbar symptoms were consistent with a suspected PCB variant. Hence, a PCB variant overlap was suspected alongside the primary MFS diagnosis. The precipitating infection causing this presentation was likely an active COVID-19 infection.

This is not the first presentation of neurological symptoms for this patient. Over the last 10 years, he had repeated episodes of gradual onset ascending symmetrical peripheral sensory symptoms likely triggered by immune provocation. His symptoms generally peak after approximately 48 hours, plateau for a few days to up to five weeks, then gradually resolve in reverse (descending) sequence over one to two weeks. The two most severe episodes have followed flu-like illnesses. On one occasion, he presented with bilateral third nerve palsy and ptosis. As these symptoms were not consistent with a diagnosis of chronic inflammatory demyelinating polyradiculoneuropathy (CIPD) or myasthenia gravis, correlating the symptoms of this presentation with his past presentations of ophthalmoplegia and bilateral ascending paralysis posed a great challenge.

Computed tomography (CT) of the brain was unremarkable. Cerebrospinal fluid (CSF) analysis, as shown in Table 2, revealed normal protein levels, cell count, and glucose levels, with negative anti-GQ1b Abs and negative anti-GAD Abs. Nerve conduction showed absent right median, ulnar, and superficial peroneal sensory responses, an amplitude drop with stimulation above the elbow, and no significant motor conduction velocity slowing. These findings were indeed in keeping with a GBS (Miller-Fisher variant).

**Table 2:** CSF analysis results. CSF, cerebrospinal fluid; NMDA, N-methyl-D-aspartate; GAD, glutamic acid decarboxylase; Abs, antibodies; mL, milliliters; µL, microliter; g, gram; L, liter; mmol, millimole; N/A, not applicable

CSF investigations	Result	Reference range
Appearance	Clear and colorless	N/A
Volume	<1.0 mL	N/A
White cells	<1/µL	N/A
Red blood cells	<1/µL	N/A
Gram film	No organisms seen	N/A
CSF protein	0.39 g/L	0.15-0.45
CSF glucose	4.1 mmol/L	2.5-5.5
CSF NMDA receptor Abs	Negative	N/A
Anti-GQ1B Ganglioside Abs	Negative	N/A
Anti-GAD Abs	Negative	N/A

During his ICU admission, the patient completed five days of IVIG, and his recovery was remarkably rapid. The patient was extubated on the fourth day of treatment after gradual weaning of ventilatory support and was discharged home on day 9 of admission. After discharge, the neurology team reviewed the patient multiple times as an outpatient. A further nerve conduction test showed complete recovery in one month with no residual weakness, and the patient returned to his normal lifestyle.

## Discussion

MFS and PCB variants are clinical diagnoses that can sometimes be backed by further testing. Only 1%-7% of GBS cases develop the Miller-Fisher form [6]. PCB variants have only been shown to represent around 3% of GBS presentations [15]. Having SARS-CoV-2 infection precipitating this combination of overlap is extremely rare, particularly in a patient with previous recurrent GBS and such a presentation has not found any previous cases in the literature. Achieving diagnosis early has presented as a significant challenge in COVID-19-related GBS and ultimately could delay lifesaving treatment.

Our patient presented with a very rare constellation of signs and symptoms that had this been the first-ever episode of GBS, diagnosing him would have been even more challenging. Ophthalmoplegia, ataxia, and areflexia are the three typical symptoms of MFS, which was partly what our patient presented with. Bickerstaff brainstem encephalitis was unlikely as the patient did not develop hypersomnolence [16]. In addition to that, his rapidly developing bulbar symptoms led to considering the possibility of PCB overlap.

Ophthalmoplegia is likely to be caused by antibodies against the GQ1b ganglioside localized on the cell surface of the para-nodal areas of cranial nerve (CN) III, IV, and VI's extramedullary region [6]. PCB variant is also thought to exhibit similar pathophysiology involving the anti-GT1a antibodies specifically targeting CN IX, and X [13]. According to the GBS classification group, serological testing is not necessary for the diagnosis of GBS variants even though it is informative [16]. Given the order of events, SARS-COV-2 infection was thought to be the most plausible cause of the patient’s presentation.

MFS has typically been correlated with elevated serum anti-GQ1b IgG levels with a negative result being unusual in non-COVID-19-related GBS. One clinical analysis of 466 patients with MFS in the pre-COVID-19 era reported an 83% positive rate in detecting anti-GQ1b IgG antibodies [17]. However, throughout the COVID-19 pandemic, the literature has consistently reported low positive rates of anti-ganglioside antibodies [3,18]. This might be due to the different immune mechanisms in which COVID-19 causes nerve damage such as cytokine storms. [18].

One meta-analysis of cases of COVID-19-induced GBS reported a serological CSF analysis showing only 9 out of 48 patients testing positive for anti-ganglioside antibodies [3]. Another systematic review reported only one out of twenty-six patients tested positive for anti-ganglioside antibodies [19]. The rise in this incidence could have possibly caused COVID-19-induced GBS to be missed in diagnosis thus reporting lower prevalence [3].

This is also significant because, without a positive result from COVID-19 PCR testing, immunological issues may worsen the longer it stays undiagnosed since virus detection by nasopharyngeal swabs declines with time. This may also have implications such as the delay of use of immunosuppressive medications as part of the therapeutic management of COVID-19, such as IL-6 inhibitors and the development of antibody therapy which may help to treat both the main viral infection and the immune-neurological logical consequences [20]. Our confirmation in diagnosis was further supported by neurophysiology testing and the rapid clinical improvement with IVIG.

The onset of GBS symptoms has also presented atypically. Our patient reported GBS symptoms presenting almost concomitantly with the onset of COVID-19 symptoms. This phenomenon has been reported distinctly in COVID-19-induced GBS whereby neurological symptoms arise within a few days and are described as an *acute para-infection*, unlike the typically known GBS which usually takes two to three weeks for neurological symptoms to appear. These reported changes in presentation gave rise to possible new theories on the development of GBS from COVID-19 such as the contribution of cytokine release [18].

In many aspects, the manner our patient's neurological symptoms appeared after contracting SARS-CoV-2 resembles those of numerous other COVID-19 instances linked to MFS that have been previously published. While some COVID-19 infections required hospitalization due to severe COVID-19 infections, most MFS cases reported were in individuals with moderate COVID-19 infections, much to our patient [21]. According to several accounts, individuals who tested positive for COVID-19 exhibited MFS-like symptoms despite never having symptomatic COVID-19, demonstrating that SARS-CoV-2 exposure may cause the syndrome to develop in any patient, regardless of the severity of their illness [22].

MRI brain is not necessary for the diagnosis of MFS, since results are often normal and anomalies are only seen in 2% of patients, however, given the rapid presentation of neurological symptoms it was useful in ruling out any evidence of stroke [6].

Immunotherapy, proper supportive care, pain management, and when necessary, breathing assistance are the major treatments for MFS. Although they have been used in the past to treat GBS or MFS, oral or IV steroids are no longer advised due to no benefits shown in multiple randomized trials [23]. Treatments for severe cases of MFS and GBS include IVIG and plasma exchange. There is no statistically significant difference between IVIG and plasma exchange when comparing main outcomes such as curative rates, relapse rates, and duration of intubation [24]. Our case demonstrates that detailed history taking and clinical examination are key in diagnosing such complex neurological disorders as such presentations can present with normal imaging and CSF analysis.

## Conclusions

Immune-mediated neurological syndromes such as GBS have been frequently reported in the literature, with a rise in the number of cases being reported after the start of the COVID-19 pandemic, including variants such as MFS. This presentation was rare due to the MFS triad of symptoms overlapping with the PCB variant, causing bulbar symptoms, with COVID-19 infection being the culprit. It is becoming increasingly important for clinicians to consider such syndromes as they can be challenging to diagnose because patients presenting with neurological symptoms and signs are not localized to one area of the brain. In addition, imaging is typically normal and CSF analysis can often be normal particularly if SARS-CoV-2 is the etiology. Early recognition through a comprehensive history, detailed neurological examination, and regular respiratory function monitoring are highly relevant as the severity of the respiratory function decline can be life-threatening. Limited data on the association between MFS and other GBS variants with COVID-19 are available in the literature. Therefore, further research is required to enhance our understanding of the pathophysiology of such an association.
